# Health Goal Attainment of Patients With Chronic Diseases in Web-Based Patient Communities: Content and Survival Analysis

**DOI:** 10.2196/19895

**Published:** 2020-09-11

**Authors:** Jiahe Song, Pei Xu, David B Paradice

**Affiliations:** 1 Haworth College of Business Western Michigan University Kalamazoo, MI United States; 2 Harbert College of Business Auburn University Auburn, AL United States

**Keywords:** web-based patient communities, self-reflection, social support, goal attainment, web-based chronic disease management, survival analysis

## Abstract

**Background:**

Activities directed at attaining health goals are a major part of the daily lives of those fighting chronic diseases. A proliferating population of patients with chronic diseases are participating in web-based patient communities, wherein they can exchange health information and pursue health goals with others virtually.

**Objective:**

In this study, we aimed to understand the effect of participation in social media–enabled web-based patient communities on health goal attainment. In particular, we studied the antecedents of health goal attainment in terms of social support and self-reflection in web-based patient communities.

**Methods:**

This data set consists of web-based health management activities of 392 patients across 13 health support groups, that is, groups with medical issues such as high blood pressure, diabetes, and breast cancer; the data of the activities were collected from a leading web-based patient community. Content analysis was used to code the social interactions among the patients on the web-based platform. Cox regression for survival analysis was used to model the hazard ratio of health goal attainment.

**Results:**

Our analysis indicated that emotional support from web-based patient communities can increase patients’ probability of achieving their goals (hazard ratio 1.957, 95% CI 1.416-2.706; *P*<.001) while informational support does not appear to be effective (*P*=.06). In addition, health-related self-reflection increases the patients’ likelihood of goal attainment through web-based patient communities (hazard ratio 1.937, 95% CI 1.318-2.848; *P*<.001), but leisure-oriented self-reflection reduces this likelihood (hazard ratio 0.588, 95% CI 0.442-0.784; *P*<.001).

**Conclusions:**

Social media–enabled web-based platforms assist health goal management via both social interaction and personal discipline. This study extends the understanding of web-based patient communities by investigating the effects of both social and cognitive factors on goal attainment. In particular, our study advocates that health goals relating to chronic conditions can be better managed when patients use the facilities of web-based health communities strategically.

## Introduction

### Background

The modern day increase in life expectancy in the global population is coincident with an increase in chronic conditions and diseases. According to a recent report by the Centers for Disease Control and Prevention, 6 in 10 adults in the United States have a chronic disease, which is the leading driver of the nation’s $3.5 trillion in annual health care costs [1]. To restrain the growing economic burden, the traditional cost-ineffective medical service model, in which health experts administer complex treatments to patients and guide them in follow-up self-care, has gradually shifted toward more collaborative, community, and individual empowerment approaches [2,3].

One such approach is community-based goal striving support on social media platforms. Health goal attainment in the face of chronic diseases has long been recognized as an important indicator of patients’ health. Based on a report, 90% of the participants used goals to manage their health, and 1 in 3 of them intended to know more about how to eventually attain their health goals [4]. With the increasing popularity of social media–enabled web-based patient communities, an increasing number of patients, especially those with chronic illnesses, are joining their peers in goal-striving activities. However, despite their popularity, the effectiveness of web-based patient communities on the self-managed goal attainment of patients with chronic diseases is largely characterized by a lack of investigation.

From a sociopsychological perspective, the outcome of a person’s health management is dependent on both social influence and self-influence [5]. During the goal-striving process in web-based patient communities, social support from peers on these web-based platforms and patients’ self-reflection are the 2 essential influencers for goal attainment. Social support functions through peer interaction on social media, which could potentially reduce patients’ uncertainty and stress. Self-reflection is a cognitive process, which offers a comparison mechanism [6,7]; therefore, the goal setter can identify what needs to be improved or what needs to be included for goal attainment. In this study, we empirically investigate the impact of social support and self-reflection on goal attainment by conducting a survival analysis on a unique data set collected from a leading web-based patient community.

### Hypotheses

#### Disease Self-Management and Web-Based Health Goal Attainment

Disease self-management is a patient-centered approach that reduces health care costs while improving the health status [8]. Such illness management is especially crucial for patients with chronic diseases as they may endure a long period of care and treatment. Most of the past research on disease self-management was related to patient education programs provided by health care professionals [9]. Internet and social media technology have made it easier to observe patients’ personal and interpersonal behaviors in web-based disease management. For instance, many web-based patient communities allow patients to monitor their goal-striving activities while developing web-based dialogues with others.

**Figure 1:**
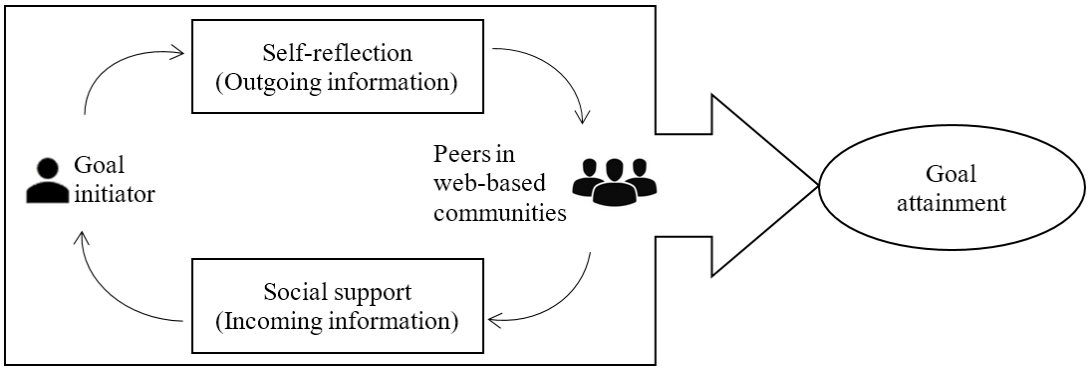
Self-reflection and social support driving goal attainment in web-based patient communities.

In health care, goal attainment is often viewed as the final completion of goals and purposes for improving one’s well-being [10,11]. Individuals can set any goal based on their health concerns, some of which are more intrinsically motivated (eg, walking goals, calorie goals) [11,12] while some are related to more extrinsic medical decisions (eg, treatment-aligned goals) [13,14]. In this study, web-based health goal attainment is the act of achieving health-beneficial goals, either fully or partially intrinsically motivated, through the use of a web-based patient community. Examples of such goals are “finish current treatment” for patients with tumors, “be emotionally stable” for patients with bipolar disorder, and “lose weight” for patients with high blood pressure. Extant studies tend to focus on the antecedent roles of goals such as the effect of a goal’s presence or absence [15] and the impact of the distance to goal attainment [15,16]. However, knowledge is lacking on goal attainment as an outcome variable. In this study, we propose a web-based goal attainment model (Figure 1) to understand the impact of social support and self-reflection on goal attainment in web-based patient communities.

#### Social Support and Goal Attainment

Social support refers to “a coping resource from which people may draw when handling stressors” [17]. In the web-based environment, social support can be exchanged between any 2 individuals with social ties. Social support can be viewed as social cognitive means in health practices [5]. In web-based patient communities, informational support and emotional support are frequently generated by patients [18].

Informational support refers to the type of assistance that helps define, comprehend, and cope with stressful problems (ie, health conditions in this research) [19,20]. Web-based informational support can be in the form of advice, referrals, or teaching [21]. According to the social judgment theory [22], one might expect that people put more weight on opinions closer to their own while discounting more distant ideas [23,24]. With regard to achieving a health goal, the support receiver (ie, the goal initiator) is likely to receive practical, health-oriented information from those who have the same health issue, if not with exactly the same goal. It can be expected that the support receiver will consider such information seriously. Research also shows that conformity in opinions is important in people’s decision-making processes. For patients, similarly, the informational support of peers is based on peers’ understanding and experience of a given medical condition, which is likely to be helpful for the goal initiator. In addition, prior research has suggested that informational support is given to a patient to reduce the uncertainty he/she is facing and to guide his/her action [25] and should be beneficial for those intending to improve their health [26]. Thus, we anticipate the goal attainment likelihood to be positively influenced by a high volume of the received informational support in web-based patient communities. Therefore, we proposed the following hypothesis.

Hypothesis 1. Web-based informational support is positively related to goal attainment by users of web-based patient communities.

Emotional support is affective and sentimental in nature and communicates love or care [26]. It is usually expressed through understanding/empathy, encouragement, affirmation/validation, sympathy, and caring/concern [21]. Although it does not contain any constructive advice or suggestion, emotional support can help patients reduce their negative feelings due to any issues or reaffirm their self-efficacy due to noticeable progress. This type of social support acts like a stimulant directly affecting the patients’ mood state and emotion. Better mood will, in turn, help patients achieve a better state of health [27]. Besides, researchers have found that emotional support has a long-lasting effect of retaining patients in web-based patient communities [18]. Due to such emotional attachments, patients are more likely to keep striving on their goals in their virtual communities. Thus, the received emotional support from web-based patient communities is likely to increase the patients’ chances in attaining their health goals, which leads to our next hypothesis.

Hypothesis 2. Web-based emotional support is positively related to goal attainment by users of web-based patient communities.

#### Self-Reflection and Goal Attainment

Self-reflection is within the umbrella of self-regulation [28] in the goal-related literature. Self-regulation is the “self-generated thoughts, feelings, and actions that are planned and cyclically adapted to the attainment of personal goals” [29]. A comparison mechanism exists in a person’s cognition to constantly reflect and evaluate his/her goal-striving activities [6,30], helping him/her move closer toward the goal. Such a comparison mechanism is self-reflection [31] through which goal progress is generated. In social cognitive theory, Bandura [32] noted that monitoring performance has little meaning to goal attainment if the comparison mechanism in the human cognitive process is lacking. For patients pursuing health goals in web-based patient communities, their self-evaluated or self-reflected content is a critical asset indicating whether these individuals continue moving their goal-striving endeavor forward.

Because web-based patient communities largely enable interpersonal activities, it is common for patients using web-based platforms to post both health-related content and leisure-oriented content. During the health goal-striving process, the former is the reflection directly related to the person’s health or goal, whereas the latter is the cognition process regarding other things in life such as recalling childhood memories, mentioning family events, and commenting on a person. In other words, leisure-oriented self-reflection largely focuses on non–health-related topics that are loosely connected to the health goal. The literature on self-regulation posits that distraction draws one’s attention away from monitoring health goals and the related behaviors, thereby causing reduced ability in goal pursuit [33]. Prior studies also indicated that successfully controlling one’s attention is beneficial for goal performance [34]. In web-based patient communities, a patient’s self-reflection content can indicate the person’s attention regulation status. Put simply, if a patient can generate more health-related reflection content, his/her goal regulation process is considered stable and his/her goal-pursuing ability is increased. Otherwise, his/her ability of goal attainment will be hindered. In light of this, we proposed the following 2 hypotheses:

Hypothesis 3. Health-related self-reflection content is positively related to goal attainment.

Hypothesis 4. Leisure-oriented self-reflection content is negatively related to goal attainment.

## Methods

### Research Context and Data Collection

We collected data from a large web-based patient community, which launched goal management in December 2007. Prior to the data collection, the website had more than 400,000 members participating in over 500 health support groups. The support groups were generated by the platform, and each group had a unique medical or distress focus, the majority of which were chronic diseases (eg, mental disorder, cancer, diabetes). This web-based patient community allowed its members to choose health goals that were closely related to their distress to manage their health. The available goals were created by a group of health care professionals hired by this web-based platform.

**Figure 2:**
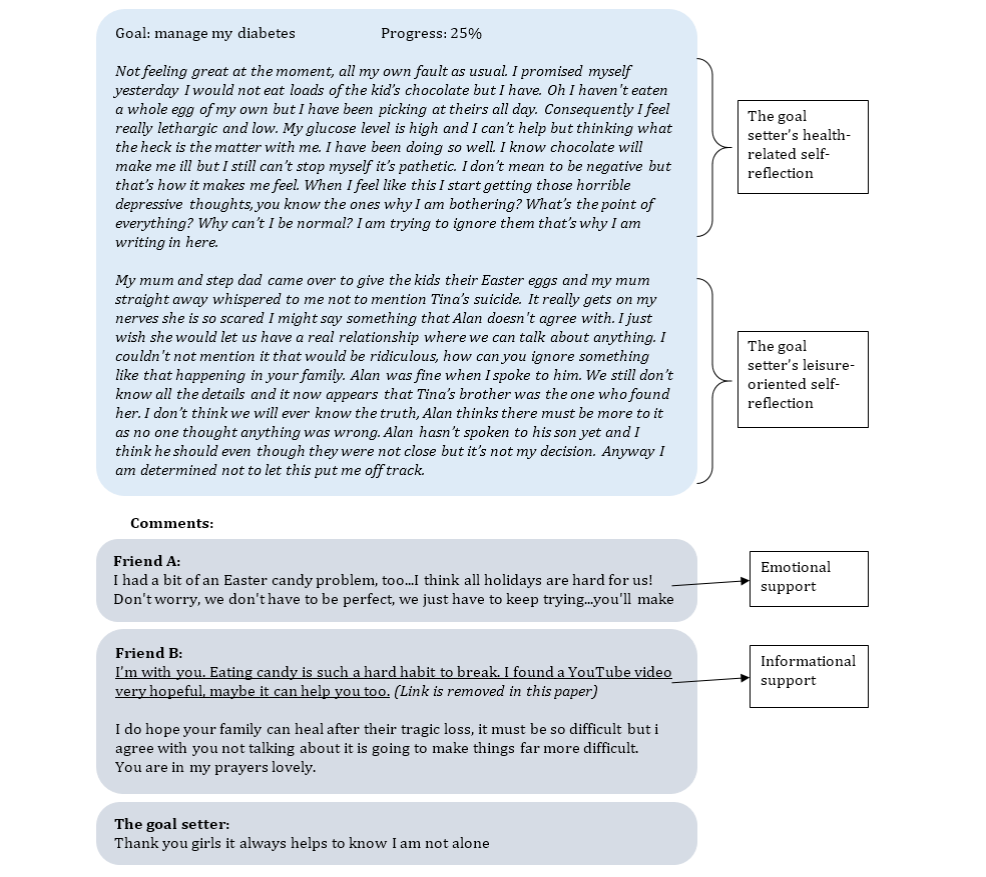
Goal progress diagram and goal update records on a patient's goal page.

At the beginning of a goal, the progress was set at 0%. Over time, patients using this web-based platform could change the progress in 5% increments. The goals were managed only by these patients. Figure 2 demonstrates a patient’s goal page. It includes a progress diagram and goal-related journals. The patient’s self-reflection content and the received social support are available to read on each journal page. We employed a web crawler to collect data from this patient community. The details of the data collection can be found in Multimedia Appendix 1. In total, 392 patients with 5 different goal types were included in the panel data set. Among them, 87 patients managed to complete their goals within the research window, while the remaining 305 left their goals unfinished.

### Variables and Content Analysis

Dependent variables: The dependent variable, *λ(t)*, is the hazard rate measuring the probability that a patient will experience goal attainment at time t. It is derived from goal duration, which represents the time spent from the goal start date to the end date. If a patient experienced health goal attainment before the end of the study period, then the end date is the actual goal end date. Otherwise, the patients are right-censored and the end date is the last day of the research window. Goal duration is assumed to have a continuous probability distribution *f(t)*. Thus, the probability that the duration will be at least *t*, *S(t)*, is defined as shown in 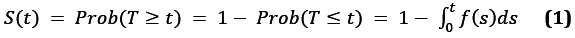. Since hazard rate is the probability that the duration will end after time t, given that it has lasted until time *t*, we define *λ(t) = f(t)/S(t)*.

Independent variables: (1) Informational represents the number of times peers in web-based patient communities provide informational support to the patient. (2) Emotional represents the number of times peers in web-based patient communities give emotional support to the patient. (3) HealthUpdate is the number of health-related contents posted in a patient’s goal update entries to measure health-related self-reflection. (4) LeisureUpdate is the number of leisure-oriented contents posted in the goal update entries to measure the leisure-oriented self-reflection. These variables were derived from a content analysis, which is presented in Multimedia Appendix 2.

Control variables: (1) Tenure indicates the length in time since a patient became a member of the web-based patient community and is measured by days. (2) NumberOfUpdate shows the total number of goal updates a patient posted until the goal attainment was censored or the end of the research window. (3) HealthResponse captures the number of patient’s health-related responses to peers in web-based patient communities in the comments section. (4) LeisureResponse captures the number of patient’s leisure-oriented responses to peers in web-based patient communities in the comments section. (5) Age in years, gender (0-male/1-female as reported by the website), and goal category dummy variables were also included. The descriptive statistics, the Pearson correlation matrix, and the variance inflation factors (VIFs) for the variables are presented in Multimedia Appendix 3. The correlations are less than 0.8, indicating that a strong correlation is not a significant concern. VIF results (mean VIF 1.74, individual VIF range 1.03-2.99) indicated that multicollinearity is not an issue in this study.

### Survival Analysis

Because the research focus is the final success in achieving health goals (ie, health goal attainment), survival and hazard ratio analysis is the appropriate approach. We used a semiparametric model, Cox proportional hazard regression, to analyze the health goal attainment data, as the predictors in the model are all continuous variables [35]. The model is specified as shown in 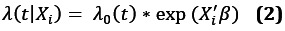. In this model, *λ_0_(t)* is a baseline hazard function that describes the risk for patients with *X_i_ = 0*, who serve as a reference cell. 
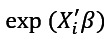
 is the relative risk, a proportionate increase or reduction in risk, associated with the set of characteristics *X_i_*. The model for this research with all the *X_i_* and control variables is shown below, where controls represents all the control variables in the research.


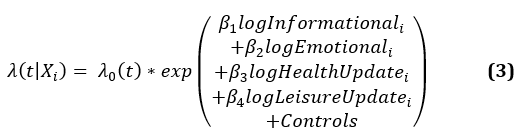


We conducted the Schoenfeld and scaled Schoenfeld residuals plots individually to check the proportionality assumption on each independent variable. *P* values greater than .05 for each term indicate that all of the terms should be kept in the model. We plotted the Cox-Snell residuals to check the goodness of fit. The plot indicated that the model fits well.

We computed the statistical power for a two-sided, .05 significance level test to detect an effect size (log hazard ratio) of 1 for each of the independent variables, given that our sample size is 392. After adjusting the censoring rate and the covariance among variables, we found that the statistical powers for the independent variables were all greater than 98%, which is well above the suggested 80% level. Thus, we conclude that our sample size is sufficient to investigate the effect of the independent variables in a Cox proportional hazard regression.

## Results

The Cox proportional hazard regression results are reported in Table 1. The results of the 4 independent variables were consistent across all 3 models, that is, the main effects were significantly related to the dependent variable in this study, except informational support. The probability of obtaining the chi-square statistic, given that there was no effect of the independent variables, taken together, on the dependent variable, was <.001 for all the 3 models.

In Hypothesis 1, we proposed that informational support boosts the chance of goal attainment for web-based patient community users. As shown in Table 1, the coefficient for logInformational is negative and not significant (*P*=.06). Thus, we did not find support for Hypothesis 1. This is probably because practical health information from nonprofessional peers in web-based patient communities does not efficiently guide one’s health goal-striving process. As shown in the results, the coefficient of logEmotional is positive and significant (hazard ratio 1.957, *P*<.001), suggesting that emotional support is positively related to goal attainment. Thus, Hypothesis 2 is supported. We used natural log for the logarithmic transformation of the variables. Thus, this result suggests that for every 3 additional emotional support comments received, the probability of goal attainment for the patient increases by 95.7%. The results show that the coefficients for health-related self-reflection and leisure-oriented self-reflection are 0.661 (hazard ratio 1.937, *P*<.001) and –0.530 (hazard ratio 0.588, *P*<.001), respectively. These findings indicate that health-related self-reflection is positively related to goal attainment, while leisure-oriented self-reflection is negatively related to goal attainment. Thus, Hypothesis 3 and Hypothesis 4 are also supported.

Two control variables, Tenure and NumberOfUpdate, are negatively related to goal attainment. For Tenure, this negative effect may be caused by the reduced interest level of older members in web-based patient communities. Over time, patients may become less motivated to use the web-based goal management features. Thus, patients with longer tenure may not frequently interact with the platform and thus delay their goal progress. Next, the negative effect of NumberOfUpdate indicates that given a fixed amount of social support and self-reflection, a patient who updates more frequently on a goal is less likely to attain the goal. In web-based patient communities, since the actual goal progress does not necessarily change in each update, frequent updating may be a result of excessive web-based self-presentation [36], which could be counterproductive to the goal attainment process [37].

**Figure 3:**
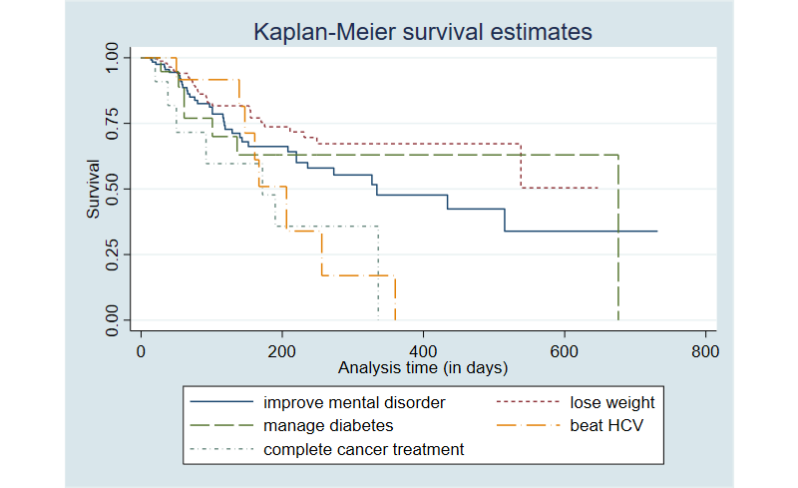
Kaplan-Meier survival estimates by goal types. HCV: hepatitis C virus.

The results also show that certain goal categories affect goal attainment. We then checked the Kaplan-Meier survival estimates graph (Figure 3) to obtain a better visualization. A test for equality of survival functions among the groups suggests that the observed differences are significant (*P*=.01). It appears that a goal may be better managed by a patient if it is highly associated with their health providers’ treatment plan (eg, beat Hepatitis C virus, complete cancer treatment). This is possibly because these goals are associated with clearer guidance, procedures, and timelines. Although beyond the scope of this study, the level of manageability of a goal due to either intrinsic factors or an extrinsic cause should be taken into account for chronic condition management.

**Table 1:** Estimates of the Cox proportional hazard model on the rate to goal attainment occurrence.

Variable	Model 1^a^ (independent variables), N=392		Model 2^b^ (control variables), n=379^c^		Model 3^d^ (full model), n=379	
	Coefficient (SE)	*P* value	Coefficient (SE)	*P* value	Coefficient (SE)	*P* value
logInformational	–0.288 (0.211)	.17	—^e^	—	–0.420 (0.220)	.06
logEmotional	0.349 (0.131)	.008	—	—	0.672 (0.165)	<.001
logHealthUpdate	0.540 (0.166)	.001	—	—	0.661 (0.197)	.001
logLeisureUpdate	–0.608 (0.133)	<.001	—	—	–0.530 (0.146)	<.001
logTenure	—	—	–1.847 (0.246)	<.001	–1.861 (0.256)	<.001
logNumberOfUpdate	—	—	–0.500 (0.240)	.04	–1.147 (0.300)	<.001
logHealthResponse	—	—	–0.255 (0.416)	.54	–0.681 (0.443)	.12
logLeisureResponse	—	—	–0.455 (0.515)	.38	–0.297 (0.542)	.58
logAge	—	—	–0.005 (0.306)	.99	–0.125 (0.336)	.71
Gender (female)	—	—	0.035 (0.312)	.91	–0.027 (0.328)	.93
Goal to lose weight	—	—	–0.574 (0.259)	.03	–0.660 (0.277)	.02
Goal to manage diabetes	—	—	0.099 (0.433)	.82	0.202 (0.483)	.68
Goal to beat Hepatitis C virus	—	—	0.816 (0.417)	.05	0.351 (0.433)	.42
Goal for cancer treatment	—	—	0.830 (0.438)	.06	0.929 (0.451)	.04

^a^Log-likelihood function, –407.5; Bayesian information criterion, 783.324.

^b^Log-likelihood function, –362.0; Bayesian information criterion, 838.935.

^c^13 values were missing for age and gender.

^d^Log-likelihood function, –334.9; Bayesian information criterion, 752.851.

^e^Not available.

## Discussion

### Principal Findings

In this study, we found that the received emotional support and health-related self-reflection in a web-based patient community increase a person’s probability of reaching a health goal. The findings also confirmed the detrimental effect of leisure-oriented content on goal attainment. We now discuss the implications for research and practice and limitations.

### Implications for Research and Practice

Our study makes several research contributions. First, this study adds new insights to health goal attainment in web-based patient communities. We conducted a survival study on the likelihood of health goal attainment with the presence of web-based social support and self-reflection. The design of the study allowed us to examine the effects of both environmental (peer support) and cognitive (self-reflection) factors on patients’ behavior related to goal pursuit. To the best of our knowledge, this is one of the first studies investigating health goal attainment in the social media setting. Second, although extant studies recognize the crucial effect of social support on patients’ health [26], it is unclear whether social support is beneficial for patients’ self-striving behaviors (eg, health goal attainment). Our study contributes to the literature by revealing the different effects of social support (ie, informational and emotional support) on patients’ goal attainment. More importantly, we found that emotional support is more beneficial than informational support in helping patients in their health goal pursuit. This means that crowd-powered positive effect may be more crucial than health information provided by the crowd (ie, those who are not health care professionals) for patients’ goal management. Additionally, this research echoes the previous research [38] on patients’ cognitive capabilities (eg, the amount of information to be processed) and pushes it further to connect it with a more specific health outcome, goal attainment, which provides building blocks for future researchers who are interested in patients’ health outcomes enabled by web-based cognitive processes.

This study also provides important practical implications. First, we suggest that patients in web-based communities setting their goals in a more specific manner as a purposeful, manageable objective are likely to benefit more from social/cognitive influencers in terms of goal attainment. Second, although setting health goals, especially health goals for chronic conditions, does not always result in winning the battle with a disease, being able to finish a goal has significant meaning in patients’ disease management and overall health. Therefore, if web-based patient communities can be integrated with offline professional health coaching methods [39], patients may realize better goal results by taking advantage of both professional-based informational support and peer-based emotional support. Lastly, if hospitals and physicians consider adding peer social interaction functionality to their web-based portals (through patient login), their patients may have a more focused and trustworthy group of peers with whom they can exchange information. Better goal performance can be expected as one of the potential benefits of using such a platform.

### Limitations and Future Research

This study has several limitations. First, we collected data from groups with active conditions to observe goal-striving activities. However, the less active groups may carry their unique characteristics that were not uncovered by our data set. Because additional desirable data are truly hard to obtain, more robustness checks may need to be conducted to help verify the results of the survival analysis. Second, although several control variables were included in this study, future studies should consider more control variables (eg, number of support givers, number of strong ties and weak ties) to reduce the omitted variable bias. Third, this study focused on goal attainment, which is the ending point of a goal. To gain more insights on goal attainment in web-based patient communities, future research can shed light on the entire process of health goal management. For example, future research can investigate whether web-based patient communities play a role in goal creation and goal progress. Such studies will help researchers and practitioners in web-based patient communities better understand how a patient’s goal proceeds over time and how to provide intervention during the process. Fourth, to the best of our knowledge, this study is among the first to empirically include patient self-reflection in web-based patient community research. An important reason for this factor to be excluded from the prior studies is that self-reflection is highly personal and cognition-oriented. Put simply, a large portion of a person’s self-reflection process for self-managed goals is not web-based and is undocumented. In this study, we were restricted to capturing only the observable portion of self-reflection through patients’ web-based activities. For future studies, multiple data sources (eg, web-based, clinical settings, interview, survey) should be implemented to reduce this issue.

## Appendix

Multimedia Appendix 1 Details about the selected goals and disease groups.

Multimedia Appendix 2 Content Analysis.

Multimedia Appendix 3 Other related statistics.

## Abbreviations

VIF: variance inflation factor
