# Supplementary material for: Health Goal Attainment of Patients With Chronic Diseases in Web-Based Patient Communities: Content and Survival Analysis
Source: J Med Internet Res. 2020 Sep 11;22(9):e19895. doi: 10.2196/19895 (PMC7519431; doi:10.2196/19895)
Supplement: Multimedia Appendix 1 [file jmir_v22i9e19895_app1.docx]

**Appendix 1.** **Details about the selected goals and disease groups.**

We conducted a simple distribution analysis on goal start and end time. We found that patients usually needed up to two years on average to complete a health goal. Thus, we collected two years of patient goal activities since the website launched its health goal management function.

We chose 13 support groups (see Table A1) to collect data for the following reasons. (1) Group members tended to read other people’s goal updates rather than managing their own initiatives. Thus, we needed groups with enough members engaging in their goal management activity. (2) Since the research focus is on health goals, we did not include family and relationship related support groups (e.g., parenting, divorce, etc.). We selected the most popular health goal within each support group as the focus goal. For example, “Lose Weight” was the most popular goal in “High Blood Pressure” and “Diets & Weight Maintenance” groups; the goal type is also relevant to members’ health in these two groups. Thus, we selected “Lose Weight” as the focus goal in these two groups. Similarly, four other types of goals were selected from the other groups. It is possible that one patient had more than one health condition and joined more than one group among these 13 groups. Thus, duplicate information was eliminated before we finalized the data set. Applying our criteria and the time window, a total of 392 patients on the web-based community were included in the panel data set. 87 patients managed to complete their goals within the research window, while the remaining 305 left their goals unfinished.

Table A1. Selected Goals and Disease Groups

| Goal Type | Support Group | Total Group Members | Total Goal Setters | Total Completed Goals | Completion Rate |
| --- | --- | --- | --- | --- | --- |
| Lose Weight | High Blood Pressure | 1464 | 163 | 13 | 8% |
|  | Diets & Weight Maintenance | 12202 | 3085 | 199 | 6% |
| Manage Diabetes | Diabetes Type I | 1488 | 89 | 5 | 6% |
|  | Diabetes Type II | 3928 | 218 | 16 | 7% |
| Beat HCV | Hepatitis C | 3693 | 122 | 39 | 32% |
| Complete Cancer Treatment | Teens with Cancer | 122 | 11 | 2 | 18% |
|  | Breast Cancer | 1323 | 44 | 5 | 11% |
|  | Colon Cancer | 763 | 33 | 4 | 12% |
|  | Lung Cancer | 865 | 17 | 1 | 6% |
|  | Brain/CNS Tumors | 851 | 17 | 2 | 12% |
| Improve Mental Disorder | Bipolar Disorder-Teen | 1506 | 92 | 9 | 10% |
|  | Bipolar Disorder-adult | 21615 | 1325 | 91 | 7% |
|  | physical & Emotional Abuse | 11340 | 672 | 61 | 9% |
| *5 Goal Types* | *13 Support Groups* | *61160* | *5888* | *447* | *8%* |
