# Supplementary material for: Health Goal Attainment of Patients With Chronic Diseases in Web-Based Patient Communities: Content and Survival Analysis
Source: J Med Internet Res. 2020 Sep 11;22(9):e19895. doi: 10.2196/19895 (PMC7519431; doi:10.2196/19895)
Supplement: Multimedia Appendix 2 [file jmir_v22i9e19895_app2.docx]

**Appendix 2. Content analysis.**

The purpose of this content analysis is to construct the explanatory variables, by coding text content harvested from the web-based patient communities. For the two aspects (i.e., informational and emotional support) under social support, we adopted Bambina’s [1] definitions and examples to guide our coding process. For self-reflection content, we guided our coding by the definitions of health-related and leisure-oriented self-reflection that we provided in the main body of the study (p.4). The definitions, detailed support examples, as well as examples quoted from the website are elucidated in the coding book (Table A2). We conducted a directed content analysis [2] to code social support categories from the collected web records by goal update. A total of 2570 records (goal updates) were coded. The coding started with a clear definition of each category. Then, two coders were involved to independently code the text, and the reliability of the content analysis was checked for confirming the results.

We followed Krippendorf’s [3] and Landis and Koch’s [4] suggestions to design the specific process in this content analysis. First, a meeting was held between two researchers (as coders) to understand and discuss the concept and definitions together. A coding book was generated during the meeting. Second, we conducted a pilot coding. 30 patients’ goals, or 197 records, were randomly selected from the data set to test whether the pilot coding result exceeds the threshold kappa value of 0.7 [3,4]. The kappa value of 0.7 indicates that the agreed understanding between coders is significantly higher than what can be concluded by chance. The coders conducted two rounds of independent coding until they reached the threshold kappa value of 0.7. During the third round, each coder coded another 40 patients’ goals (248 records), and the result exceeded the kappa value of 0.7. This pilot coding confirmed that the coding book is robust. Third, the two coders separately coded the rest of the data set (2125 records). Last, all the codes were documented in the panel data set for the data analysis stated in the next section.

Table A2. Coding book for content analysis.

| Independent Variables | Definition |
| --- | --- |
| **Informational support** | Informational support is provided to the type of assistance that helps define, comprehend, and cope with stressful problems (i.e., health problems in this research). Examples of this support type: advice, referral, and teaching [1]. |
| *Examples in the web-based patient communities:*   1. “I was first put on Femara. However, I had to switch to Tamoxifen as I could not tolerate the side effects of the Femara…” 2. “I suggest you look into “Zone.” Eat 3 meals daily + 2 snacks, just avoid bad carbs and sugar.” 3. “It (losing weight) takes time, determination, and of course dedication, eating healthy and exercise).” | |
| **Emotional support** | Emotional support is affective and sentimental in nature and communicates love or caring. Examples of this support type: understanding/empathy, encouragement, affirming/validation, sympathy, and caring/concern [1]. |
| *Examples in the web-based patient communities:*   1. “I went to bed at 2:30AM and was up at 7 so I know how you feel. Isn’t bipolar FUN??? LOL!” 2. “Hugs!!!” 3. “Gosh, that’s a lot to put on your shoulders, especially when you need direction and advice.” 4. “You’ve made the right choice! Just as this journey is different for each of us, so are the decisions we have to make [… ]” 5. “I just want you to know that don’t think it’s you ... it’s something in him ... please don’t take it personally […]” | |
| **Health-related self-reflection** | This is the self-reflection directly related to the person’s health and/or the health goal. Examples of this reflection type: talking about recent diet, medicine, physical activity, or doctor visit. |
| *Example in the web-based patient communities:*  “I did my 30 min on the treadmill and 10 sit up (so far, more to come), 40 push-ups too!”  “I've lost 13 pounds total, but only 3.5 since I started my diet for the New Year. “ | |
| **Leisure-oriented self-reflection** | This is the cognition process regarding other things (not focused on health topics) in life. Examples of this reflection type: recalling childhood memories, mentioning family events, or commenting on a person. |
| *Example in the web-based patient communities:*  “I am having a quiet weekend as usual. Yesterday I finally picked up my car. It look great. Sure felt great being behind the wheel again.”  “I just don't like cold weather. More than 50 years away from living in the Islands and I still can't get used to the cold weather.” | |

**References**

1. Perloff, R.M. The dynamics of persuasion: communication and attitudes in the 21st century (4th ed.); 2010; New York, NY: Routledge. ISBN: 0415805686
2. Hsieh, H.F., Shannon, S.E. Three approaches to qualitative content analysis. Qualitative Health Research; 2005; 15(9):1277-1288. DOI: [10.1177/1049732305276687](https://doi.org/10.1177/1049732305276687)
3. Krippendorff, K. (2012). Content analysis: an introduction to its methodology (3^rd^ ed.). Thousand Oaks, CA: Sage; 2012. ISBN: 1412983150
4. Landis, J.R., Koch, G.G. The measurement of observer agreement for categorical data. Biometrics; 1977; 33(1):159-174. DOI: [10.2307/2529310](https://www.jstor.org/stable/2529310)
