# Supplementary material for: Health Goal Attainment of Patients With Chronic Diseases in Web-Based Patient Communities: Content and Survival Analysis
Source: J Med Internet Res. 2020 Sep 11;22(9):e19895. doi: 10.2196/19895 (PMC7519431; doi:10.2196/19895)
Supplement: Multimedia Appendix 3 [file jmir_v22i9e19895_app3.docx]

**Appendix 3. Other related statistics.**

Table A3. Descriptive Statistics (N=392)

| Variable | Mean | S.D. | Min | Max |
| --- | --- | --- | --- | --- |
| logInformational | 0.519 | 0.748 | 0.000 | 3.871 |
| logEmotional | 1.132 | 1.163 | 0.000 | 5.153 |
| logVerbalUpdate | 1.348 | 0.736 | 0.000 | 3.970 |
| logLeisureUpdate | 1.026 | 0.813 | 0.000 | 4.094 |
| logTenure | 4.556 | 1.458 | 0.000 | 7.281 |
| logNumberOfUpdate | 1.539 | 0.555 | 0.693 | 4.248 |
| logHealthResponse | 0.117 | 0.344 | 0.000 | 2.398 |
| logLeisureResponse | 0.111 | 0.324 | 0.000 | 2.079 |
| logAge | 3.445 | 0.367 | 2.398 | 4.635 |
| gender | 0.881 | 0.324 | 0.000 | 1.000 |

Table A4. Pearson Correlation Matrix

|  | Variable | 1 | 2 | 3 | 4 | 5 | 6 | 7 | 8 | 9 | 10 |
| --- | --- | --- | --- | --- | --- | --- | --- | --- | --- | --- | --- |
| 1 | logInformational | 1.00 |  |  |  |  |  |  |  |  |  |
| 2 | logEmotional | 0.79^***^ | 1.00 |  |  |  |  |  |  |  |  |
| 3 | logVerbalUpdate | 0.54^***^ | 0.54^***^ | 1.00 |  |  |  |  |  |  |  |
| 4 | logLeisureUpdate | 0.34^***^ | 0.40^***^ | 0.48^***^ | 1.00 |  |  |  |  |  |  |
| 5 | logTenure | 0.16^**^ | 0.24^***^ | 0.27^***^ | 0.21^***^ | 1.00 |  |  |  |  |  |
| 6 | logNumberOfUpdate | 0.37^***^ | 0.38^***^ | 0.55^***^ | 0.44^***^ | 0.45^***^ | 1.00 |  |  |  |  |
| 7 | logHealthResponse | 0.33^***^ | 0.27^***^ | 0.21^***^ | 0.11^*^ | 0.07 | 0.21^***^ | 1.00 |  |  |  |
| 8 | logLeisureResponse | 0.24^***^ | 0.28^***^ | 0.12^*^ | 0.23^***^ | 0.12^*^ | 0.24^***^ | 0.57^***^ | 1.00 |  |  |
| 9 | logAge | 0.20^***^ | 0.23^***^ | 0.09 | 0.12^*^ | 0.17^***^ | 0.06 | 0.17^***^ | 0.22^***^ | 1.00 |  |
| 10 | gender | -0.04 | -0.03 | 0.01 | -0.03 | -0.12^*^ | -0.09 | -0.03 | -0.02 | -0.01 | 1.00 |

^*^ *p* < 0.05, ^**^ *p* < 0.01, ^***^ *p* < 0.001

Table A5. VIF Results for Multicollinearity

| Variable | VIF | SQRT VIF | Tolerance | R-Squared |
| --- | --- | --- | --- | --- |
| logInformational | 2.94 | 1.71 | 0.3402 | 0.6598 |
| logEmotional | 2.99 | 1.73 | 0.3350 | 0.6650 |
| logVerbalUpdate | 2.01 | 1.42 | 0.4976 | 0.5024 |
| logLeisureUpdate | 1.49 | 1.22 | 0.6698 | 0.3302 |
| logTenure | 1.32 | 1.15 | 0.7549 | 0.2451 |
| logNumberOfUpdate | 1.83 | 1.35 | 0.5467 | 0.4533 |
| logHealthResponse | 1.64 | 1.28 | 0.6097 | 0.3903 |
| logLeisureResponse | 1.66 | 1.29 | 0.6007 | 0.3993 |
| logAge | 1.17 | 1.08 | 0.8521 | 0.1479 |
| gender | 1.03 | 1.01 | 0.9721 | 0.0279 |
| goalcategory | 1.08 | 1.04 | 0.9261 | 0.0739 |
| Mean VIF | 1.74 |  |  |  |
